# Complement Markers in Blood and Urine: No Diagnostic Value in Late Silent Antibody-Mediated Rejection

**DOI:** 10.1097/TXD.0000000000000915

**Published:** 2019-06-27

**Authors:** Blanka Mező, Andreas Heilos, Georg A. Böhmig, Farsad Eskandary, Markus Wahrmann, Gregor Bond, Nicolas Kozakowski, Philip F. Halloran, Krisztina Rusai, Zoltán Prohászka

**Affiliations:** 1 IIIrd Department of Internal Medicine and MTA-SE Research Group of Immunology and Hematology, Research Laboratory, Hungarian Academy of Sciences and Semmelweis University, Budapest, Hungary.; 2 Department of Pediatrics and Adolescent Medicine, Medical University of Vienna, Vienna, Austria.; 3 Division of Nephrology and Dialysis, Department of Medicine III, Medical University of Vienna, Vienna, Austria.; 4 Department of Clinical Pathology, Medical University of Vienna, Vienna, Austria.; 5 Alberta Transplant Applied Genomics Centre, ATAGC, University of Alberta, Edmonton, AB, Canada.

## Abstract

**Background.:**

Antibody-mediated rejection (AMR) is a major cause of kidney allograft failure. Its molecular mechanisms are multifaceted and may include a role of complement activation via the classical pathway. Here, we investigated whether noninvasive complement monitoring adds predictive power to the diagnosis of AMR in the setting of donor-specific antibody (DSA) positivity.

**Methods.:**

In this cross-sectional study, 741 kidney transplant recipients with stable graft function ≥180 days posttransplantation were screened for the presence of human leukocyte antigen (HLA) alloantibodies. Eighty-three of 111 DSA-positive recipients underwent protocol biopsies and were tested for blood and urinary levels of complement proteins (C1q, C4, C3) and activation products (C4d, C3a, C5a, C5b-9).

**Results.:**

Forty-seven recipients were diagnosed with AMR, and 21 were C4d-positive. While biopsy-confirmed AMR (and C4d) associated with DSA-binding strength (IgG mean fluorescence intensity of the immunodominant DSA versus AMR; area under the receiver operating characteristic curve: 0.76), tested complement markers did not have any predictive value for rejection (area under the receiver operating characteristic curve: 0.49–0.56). There were, however, tight correlations between complement activation products in urine and protein/creatinine ratio (*ρ* = 0.44–0.64; *P* < 0.001). Analysis of death-censored graft survival over a median of 60 months revealed no independent associations with levels of complement markers in blood or urine.

**Conclusions.:**

Complement patterns in blood and urine failed to identify AMR in late biopsies and may have no relevant diagnostic value in this particular context.

## INTRODUCTION

Antibody-mediated rejection (AMR) is one of the cardinal causes of graft dysfunction and failure in kidney transplantation.^1^ This type of rejection—commonly triggered by human leukocyte antigen (HLA) antibodies—is characterized by ongoing inflammation in the microcirculation that may progress to irreversible injury.^1^ Serological (detection of donor-specific antibodies [DSA]), morphological (inflammation/injury in peritubular and glomerular capillaries, capillary C4d deposition), and molecular diagnostic criteria are now well defined,^2,3^ but the treatment of AMR is still a major challenge.^4-6^

Detection of circulating DSA is key to the diagnosis of AMR.^2^ Nevertheless, DSA detection may not necessarily implicate an ongoing rejection process, and it was shown that some DSA-positive recipients maintain stable graft function over long periods of time.^7^ Cohort studies have shown that de novo DSA formation associates with a progressive decline in estimated glomerular filtration rate (eGFR).^8^ In patients without graft dysfunction at the time of DSA occurrence, however, the effect on eGFR slope was found to be less pronounced, and some of the patients did not show any rejection features.^8^ Moreover, in a cross-sectional screening study—performed in the context of an interventional trial to assess the effect of bortezomib in late AMR (BORTEJECT trial)—we found that among DSA-positive patients, only every second was diagnosed with AMR.^9^ These data reinforce the need for allograft biopsies to confirm a pathogenetic role of detected DSA.

A better understanding of the molecular mechanisms of DSA-triggered graft injury may provide clues to the establishment of noninvasive rejection biomarkers. The pathophysiology of AMR is multifaceted and may include a contribution of a variety of complement-dependent and -independent (Fc gamma receptor-triggered) mechanisms.^10-12^ Indirect evidence for a pathophysiological role of classical pathway (CP) complement activation comes from the finding of capillary C4 split product C4d deposition in a subset of AMR cases, a feature tightly related to adverse transplant outcomes.^13,14^ In addition, serological detection of complement-fixing (when compared with non complement-fixing) DSA in single antigen bead assays, mainly reflecting high levels of DSA binding, was found to be associated with inferior transplant outcomes.^15^

Given the presumed pathogenetic role of intra-graft CP activation, a potential noninvasive strategy to dissect the clinical relevance of a given HLA antibody pattern may be the detection of CP function and complement products in peripheral blood or urine. Indeed, in an earlier study, detection of CP split product C4d in urine (but not C5b-9) was found to be associated with capillary C4d staining and rejection.^16^ These results, however, were not confirmed in a subsequent study, and urinary C4d excretion was interpreted as a marker of unspecific glomerular injury.^17^ Such controversial results may have been due to small sample sizes or differences in case selection and rejection criteria. Moreover, one may argue that distinct CP activation markers reflecting activation of defined steps within the cascade may subtly differ in their diagnostic sensitivity and specificity. Furthermore, as diverse events like ischemia/reperfusion, rejection, or glomerulonephritis may activate the CP, the sole detection of complement markers may not be able to dissect reliably the pathophysiology of allograft dysfunction.

The primary objective of the present study was to determine whether the detection of complement components and split products in the blood and urine of DSA-positive kidney transplant recipients is able to predict AMR in concomitant protocol biopsies. Our study was performed in the context of the cross-sectional screening phase of the BORTEJECT trial,^4^ which included an in-depth analysis of DSA characteristics and morphological/molecular AMR features.

## MATERIALS AND METHODS

### Study Design and Patients

**FIGURE 1.**
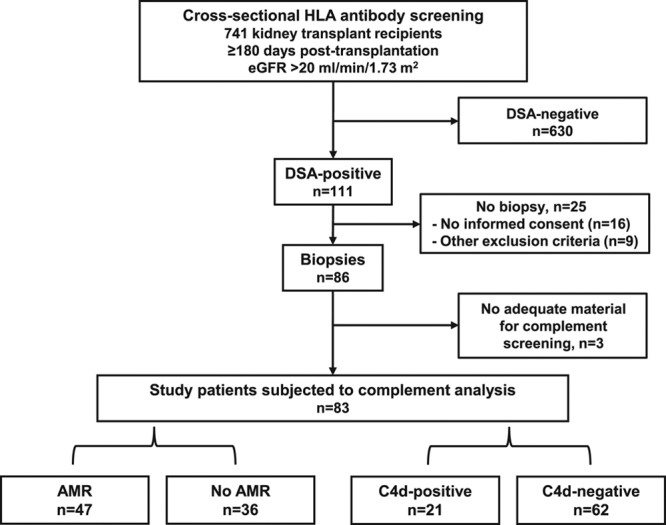
Study flow chart. HLA antibody screening of 741 kidney transplant recipients led to the identification of 111 DSA-positive recipients. Eighty-six DSA-positive patients underwent protocol biopsies. For 83 subjects (study patients), adequate material for complement screening was available. Of those, 47 had AMR, and 21 showed capillary deposits of C4d. AMR, antibody-mediated rejection; DSA, donor-specific antibody; eGFR, estimated glomerular filtration rate; HLA, human leukocyte antigen.

The present study was based on a prospective cross-sectional AMR screening in the context of the BORTEJECT trial (www.clinicaltrials.org: NCT01873157), which was designed to assess the effect of bortezomib on the course of late AMR.^4,18^ The trial was approved by the institutional ethics committee of the Medical University of Vienna (EK1515/2012) and conducted in accordance with the Declarations of Helsinki and Istanbul. During the screening period (between October 2013 and February 2015), 1165 kidney transplant recipients were registered at the Vienna Transplant Unit (outpatient clinic of the nephrology department) (Figure 1). In total, 1076 patients fulfilled 3 key inclusion criteria: (1) age >18 years, (2) stable allograft function after ≥180 days posttransplantation, and (3) a Mayo Clinic eGFR^19^ >20 mL/min/1.73 m^2^. Of these, 741 patients underwent anti-HLA antibody prescreening. One hundred eleven patients (15%) were classified as DSA-positive and, following the study protocol, 86 of these patients underwent protocol biopsies. Twenty-five patients had no biopsy, either because of a lack of consent or the presence of one or more exclusion criteria defined in the protocol.^4^ For 83 of the 86 patients subjected to biopsy, adequate material for detailed evaluation of complement products in both peripheral blood (serum or plasma) and urine was available (Figure 1). Material was collected and stored (in parallel to prospective DSA testing) 23 days (median; interquartile range [IQR]: 15–42 days) before protocol biopsies. Blood, urine, and biopsies were in all subjects obtained before therapeutic interventions within or outside the BORTEJECT trial (treatment with bortezomib vs placebo). Baseline characteristics of the 83 study patients are summarized in Table 1.

**TABLE 1.**
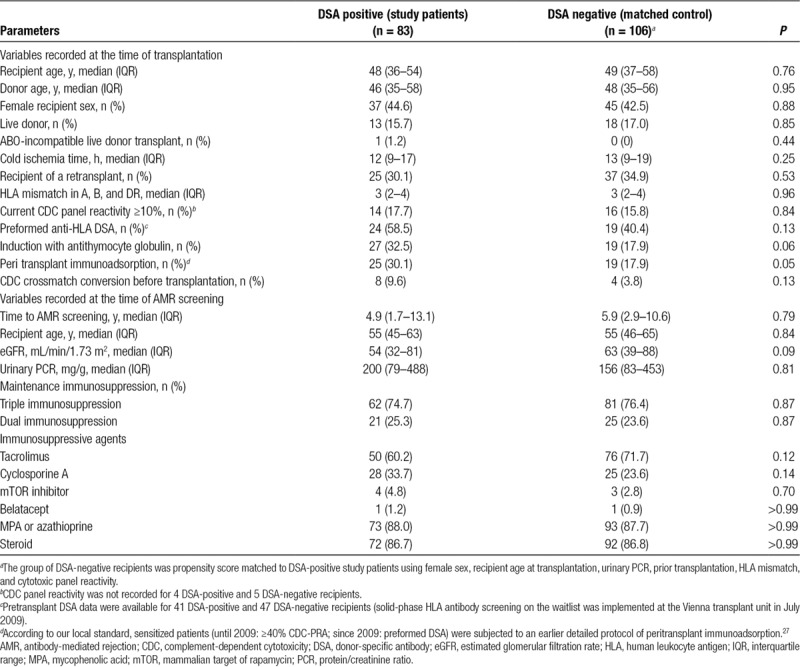
Baseline demographics and patient characteristics

For comparative analyses in relation to DSA status, 106 of the 630 DSA-negative patients were propensity score matched with the DSA-positive study patients using female sex, recipient age at transplantation, protein/creatinine ratio (PCR), prior transplantation, HLA mismatch, and cytotoxic panel reactivity. With the exception of more frequent desensitization among DSA-positive patients, groups did not significantly differ with respect to baseline data and immunosuppressive therapy (Table 1).

### Biopsies

All 83 DSA-positive study patients underwent protocol biopsies, in median 23 days (IQR, 15–41 days) after antibody screening. AMR-typical lesions, including C4d in peritubular capillaries (PTC), glomerulitis, peritubular capillaritis, and transplant glomerulopathy (cg), were defined and scored following the Banff 2013 scheme.^20^ For immunohistochemical C4d-staining on formalin-fixed paraffin-embedded sections, we used a polyclonal anti-C4d antibody (BI-RC4D; Biomedica, Vienna, Austria). Multilayering of basement membranes of PTC and glomerular basement membranes was assessed by electron microscopy. For 3 of the 83 biopsies, no ultrastructural work-up was available. One of these biopsies fulfilled none of the histomorphological and immunohistochemical criteria of AMR, and 2 were classified as chronic active AMR with moderate or high grade cg. As previously described in detail,^21^ a proportion of 1 biopsy core was stabilized in RNALater and shipped to the Alberta Transplant Applied Genomics Centre for microarray-based gene expression analysis (Molecular Microscope Diagnostic System). A classifier related to AMR was generated using a recently published reference set of 1208 biopsy specimens.^22^ AMR was defined following the Banff 2013 consensus,^20^ based on histomorphological, immunohistochemical (C4d), ultrastructural (high-grade multilayering of basement membranes of PTC), and serological (DSA detection) criteria, as well as a thoroughly validated molecular classifier for AMR (Molecular Microscope Diagnostic System molecular AMR score ≥0.2), respectively. The latter differed significantly between AMR (median: 0.62 [IQR: 0.36–0.86]) and no AMR cases (0.06 [IQR: 0.02–0.13] *P* < 0.001).

### Antibody Detection

HLA reactivity patterns were analyzed using LABScreen Single Antigen assays (One Lambda, A Thermo Fisher Scientific Brand, Canoga Park, CA) as earlier described in detail.^9^ Before alloantibody testing, serum samples were heat-inactivated (30 min, 56°C) to prevent complement interference. The presence or absence of IgG type DSA (mean fluorescence intensity [MFI] threshold >1000) was determined in the context of serological and low- or high-resolution donor/recipient HLA typing (HLA-A, -B, -Cw, -DR, -DQ, and/or -DP). For assessment of the C1q-binding ability of DSA, C1qScreen assays (One Lambda) were used. For analysis of C4 or C3 activation and fixation (deposition of C4 and C3 split products to the bead surface),^9^ beads were first incubated with patient sera followed by an excess of serum obtained from a nonsensitized healthy volunteer (complement source). Beads were then washed and incubated with biotin-conjugated monoclonal antibodies against human C4d or C3d (Quidel, San Diego, CA), and in a second step, phycoerythrin-conjugated Streptavidin. Test results were documented as the MFI of the immunodominant DSA (MFI_max) or the sum of DSA MFI (MFI_sum).

### Detection of Complement Components

All samples were collected, handled, and stored at −80°C (Biobank of the Medical University of Vienna) following a uniform predefined protocol. To exclude any batch effects, individual complement measurements were performed using uniform batches of test kits. Individual complement assays were performed at a single time point for all included subjects. Samples were randomly allocated on the plates. Total complement activity of the CP (50% hemolytic complement activity [CH50] assay) in serum samples was determined by an in-house hemolytic-titration assay based on Mayer’s method (reference range: 48–103 U/mL).^23^ An enzyme-linked immunosorbent assay was applied to measure C1q serum concentrations^24^ (reference range: 60–180 mg/L). Serum concentrations of complement C3 (reference range: 0.9–1.8 g/L) and C4 (reference range: 0.15–0.55 g/L) were determined by turbidimetry (Beckman Coulter, Brea, CA). Complement activation split product C3a (MicroVue C3a-desArg EIA, A032), C4d (MicroVue C4d EIA, A008), C5a (MicroVue C5a EIA, A025), and terminal pathway activation complex sC5b-9 (MicroVueSC5b-9 Plus EIA, A029) levels were determined with commercial kits (Quidel, San Diego, CA), according to the manufacturer’s instructions in ethylenediaminetetraacetic acid (EDTA) anticoagulated plasma or mid-stream urine samples. The methods, handling, and preparation of the urine samples were detailed previously.^25,26^ In brief, fresh urine was mixed (9:1 ratio) with 10 mM Tris buffer, pH 8.6, with 0.05% Tween 20 and 0.01% of NaN_3_ containing protease inhibitors (10 mM benzamidine, 10 mM ϵ-aminocaproic acid, 20 mM EDTA, and 100 kallikrein inhibitor units of aprotinin). After centrifugation (1890 *g*; 10 min; room temperature). Supernatants were aliquoted and stored at −80°C and thawed only once before testing. For analysis of levels in urine, complement protein concentrations were corrected according to urinary creatinine concentration. Blood samples were processed immediately, and separated serum and EDTA plasma aliquots were stored at −80° C until use. For the complement assays employed in the present study, intra-assay and inter-assay variation coefficients were <7% and <17%, respectively.

### Statistical Analysis

Continuous data were expressed as the median and IQR (range from the 25th to the 75th percentile) and categorical variables as absolute and relative frequencies. Fisher exact tests were used to compare categorical data and Mann-Whitney *U* or Kruskal-Wallis tests to compare continuous data, while nonparametric test was used for correlation analysis. For comparative analyses in relation to DSA status, propensity score matching was applied to create a matched DSA-negative control group. Variables were selected using a logit model. Variables with a *P* value <0.10 and/or confounding factors known to be associated with AMR were selected (female sex, recipient age at transplantation, number of prior transplantations, HLA mismatch, cytotoxic panel reactivity, and PCR). One-to-one matching was conducted, and balance between the final groups was assessed by propensity score distribution and descriptive statistics. The nonparametric bootstrap method, resampling with replacement 1000 times on the matched dataset, was conducted to provide inner validation. Receiver operating characteristic (ROC) curves and their corresponding area under the curve (AUC) values were calculated to assess the sensitivity and specificity of different predictors. Kaplan-Meier analysis was applied for calculation of death-censored graft survival. Mantel-Cox Log-rank test was used for comparison of survival between groups. For bivariate analysis, Cox regression analysis was performed using the following confounders: detection of urinary C4d and PCR. Hazard ratios (HRs) are presented with 95% confidence intervals (95%CI). A 2-sided *P* value <0.05 was considered statistically significant. For statistical analysis, IBM SPSS Statistics 24 (IBM Corporation, Armonk, NY) was used.

## RESULTS

### Baseline Characteristics

The study population included 83 DSA-positive kidney transplant recipients who underwent protocol biopsies after a median of 4.9 years posttransplantation. Characteristics of the DSA-positive study population (and a propensity score matched cohort of 106 DSA-negative recipients) are summarized in Table 1. Forty-five percent of the patients were females, and 16% were recipients of a live donor transplant. Twenty-five recipients were presensitized and had been subjected to an earlier described desensitization protocol of peritransplant immunoadsorption^27^ (cytotoxic crossmatch conversion in 8 patients). At the time of cross-sectional screening, 62 recipients were on triple and 21 on dual immunosuppression. Fifty patients were on tacrolimus-based immunosuppression and 72 recipients on steroids. Median eGFR was 54 mL/min/1.73 m^2^ and urinary PCR 200 mg/g (Table 1). Forty-seven patients had biopsy-confirmed AMR (C4d-positive: n = 21) as defined by the Banff 2013 scheme (acute/active AMR: n = 13; chronic active AMR: n = 32; cg without evidence of current/recent antibody interaction [C4d staining or at least moderate microvascular inflammation]: n = 2). Thirty-six recipients did not fulfill the diagnosis of AMR and, in most of these cases, no specific lesions (n = 27) were found. Two specimens showed C4d staining without morphological evidence of rejection, 3 Banff borderline rejection, and 4 glomerulonephritis (IgA nephropathy: n = 2, nonspecified phenotype of immune complex glomerulonephritis: n = 1; focal segmental glomerulonephritis: n = 1).

### Blood and Urine Complement Profile in Relation to DSA and Biopsy Results

**FIGURE 2.**
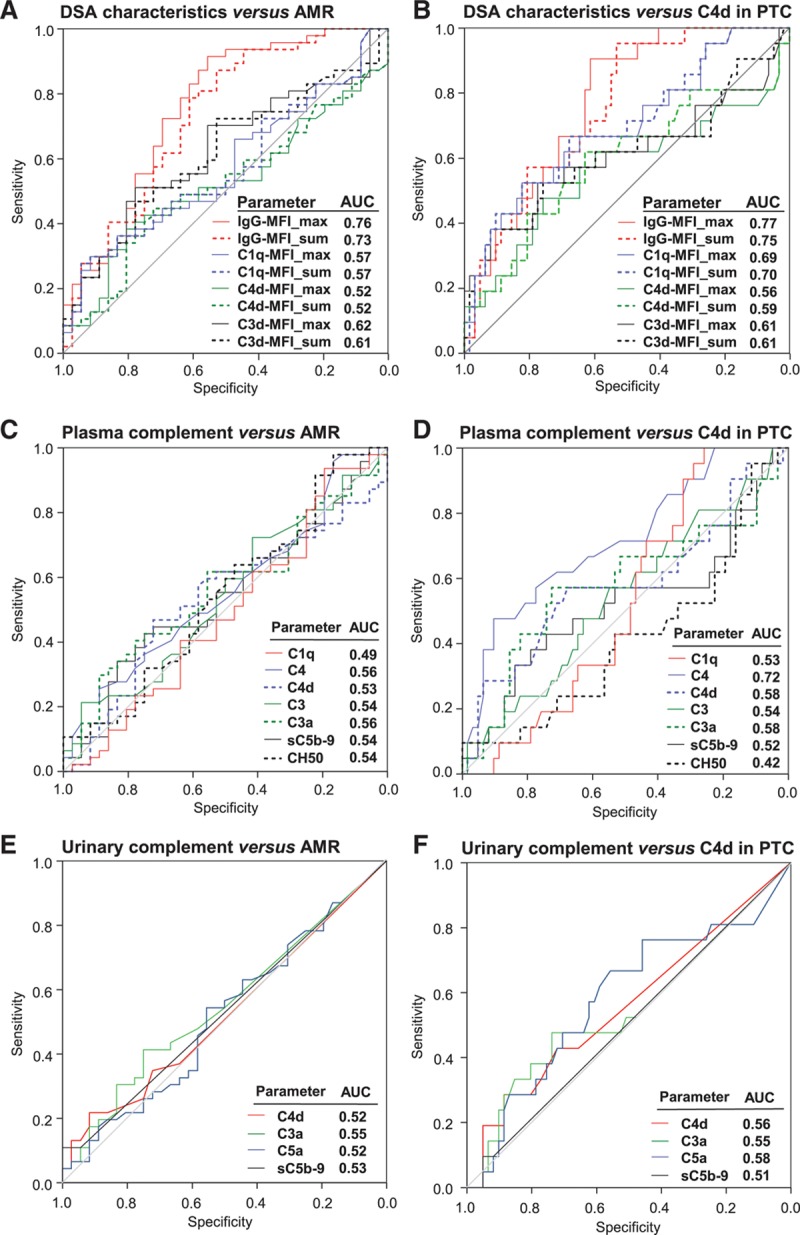
Prediction of biopsy results by DSA characteristics and complement markers. ROC curve analysis of DSA characteristics (A,B), CH50 and complement markers in peripheral blood (C,D), or creatinine-adjusted complement products in urine (E,F) was performed in comparison with the finding of biopsy-confirmed AMR (A,C,E) or C4d staining in PTC, respectively (B,D,F). Panels show ROC curves and corresponding AUC values for each comparison. AMR, antibody-mediated rejection; AUC, area under the curve; CH50, 50% hemolytic complement activity; DSA, donor-specific antibody; MFI, mean fluorescence intensity; PTC, peritubular capillaries; ROC, receiver operating characteristic.

Patients were tested for overall CP activity (CH50), plasma/serum levels of C1q, C4, C4d, C3, C3a, and sC5b-9, and concentrations of creatinine-adjusted urinary complement activation products C4d, C3a, C5a, and sC5b-9, respectively. As shown in Table 2, complement levels were not different between the 83 DSA-positive study patients and a matched group of 106 DSA-negative recipients. Furthermore, in the cohort of DSA-positive patients, we found no differences in relation to biopsy-confirmed AMR or C4d deposition (Tables 2 and 3). As illustrated in Figure 2, ROC analysis of the tested markers revealed no relevant predictive accuracy (AUC: ≤0.72).

**TABLE 2.**
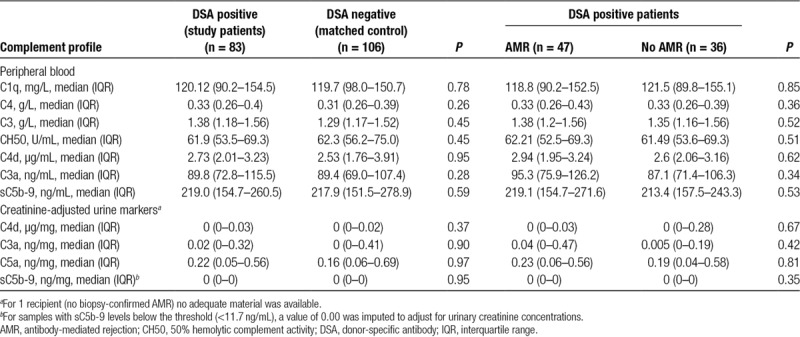
Blood and urine complement profile in relation to DSA and AMR status

**TABLE 3.**
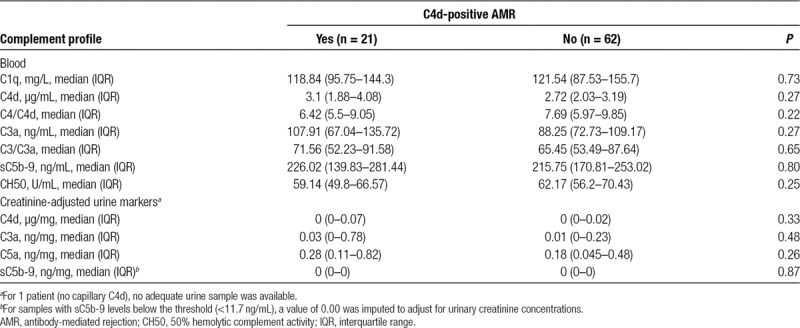
Blood and urine complement profile in relation to the finding of C4d-positive AMR in protocol biopsies

### DSA Characteristics and Blood or Urine Complement Profile

As detailed in Table 4, patients with biopsy-confirmed AMR had higher DSA levels than patients without rejection (median levels of IgG-MFI_max: 3688 vs 1491; IgG-MFI_sum: 4555 vs 1879; *P* < 0.0001). Groups, however, did not differ with respect to HLA class specificity and complement fixing capability of DSA in C1q, C4d, or C3d bead assays (Table 4). The test performance of DSA characteristics in diagnosis of AMR is shown in Figure 2. While IgG-MFI had moderate predictive power (AUC for MFI_max: 0.76; MFI_sum: 0.73), the performance of complement fixation assays was weak (AUC: 0.52–0.62) (Figure 2).

**TABLE 4.**
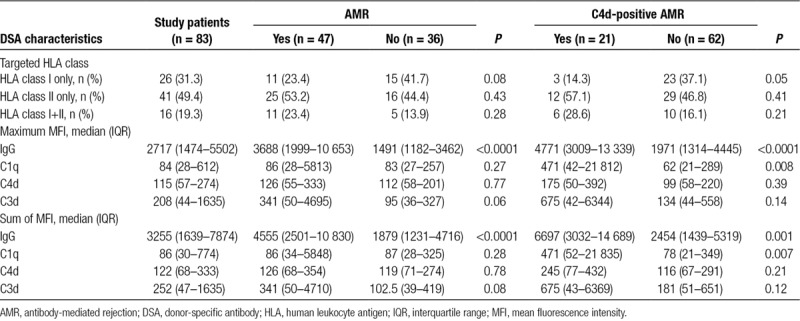
Donor-specific antibody DSA in relation to biopsy results

IgG MFI_max (median: 4771 vs 1971; *P* < 0.0001) and IgG MFI_sum (6697 vs 2453; *P* < 0.0001) were significantly higher in C4d-positive than in C4d-negative patients. In parallel, levels of C1q (but not C4d or C3d) fixation were significantly different (Table 4). In ROC analysis, IgG MFI_max had superior predictive accuracy (AUC: 0.77; complement fixation, AUC: 0.56–0.70).

Analyzing blood and urinary complement markers in relation to DSA strength and complement-fixing capability (Table 5), we found a marginal inverse correlation between overall plasma CP complement activity (CH50) and IgG MFI_max (*r*: −0.22, *P* = 0.05) but no significant correlations for C1q-, C4d-, and C3d-fixation (*ρ* = −0.2, *P* = 0.07, *ρ* = −0.19, *P* = 0.06, and *ρ* = −0.21, *P* = 0.09, respectively). Concentrations of other tested complement (split) products in blood or urine did not correlate with DSA characteristics (Table 5).

**TABLE 5.**
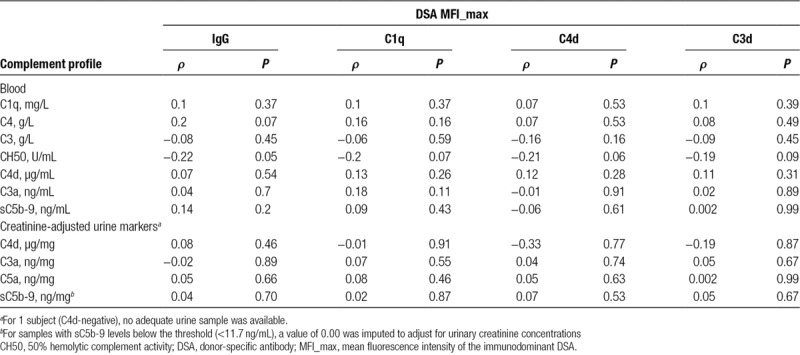
Correlation of DSA characteristics with complement markers in blood and urine

### Urine Complement Profile and Proteinuria

**FIGURE 3.**
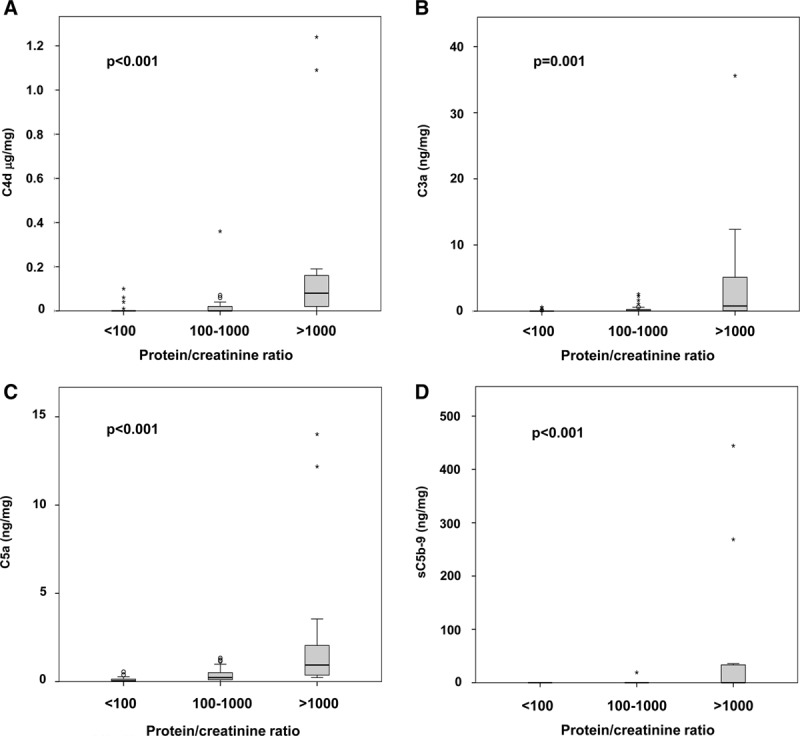
Urinary complement markers and proteinuria. Complement markers adjusted for urinary creatinine were analyzed in relation to 3 different categories defined according to the height of PCR. Box plots indicate median, IQR, and range. Mild outliers are indicated as open dots and extreme outliers as asterisks. For statistical comparisons, Kruskal-Wallis test was used. IQR, interquartile range; PCR, protein/creatinine ratio.

Creatinine-adjusted urinary C4d, C3a, C5a, and sC5b9 levels were found to correlate tightly with PCR (C4d: *ρ* = 0.38, *P* < 0.0001; C3a: *ρ* = 0.44, *P* < 0.0001; C5a: *ρ* = 0.64, *P* < 0.0001; sC5b9: *ρ* = 0.45, *P* < 0.0001), showing a stepwise increase of complement levels with increasing proteinuria (Figure 3). PCR was tightly associated with cg (487 [155–1695] vs 126 [64–239] mg/g in patients with vs without cg; *P* < 0.0001) (data not shown).

### Blood or Urine Complement Profile and Graft Survival

**FIGURE 4.**
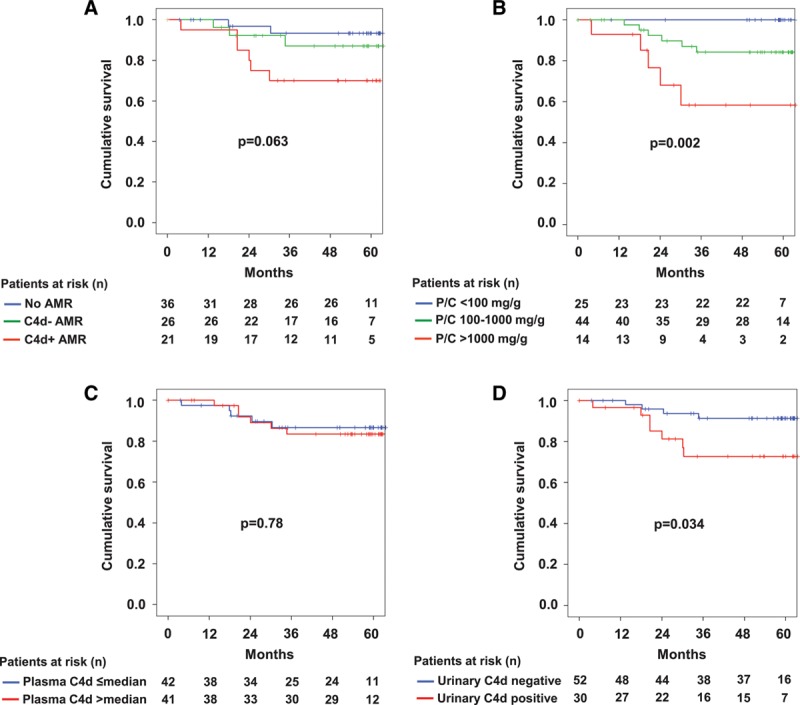
Death-censored graft survival according to biopsy results (A), urinary protein/creatinine (P/C) ratio (B), C4d in blood (C), and C4d detection in urine (D). The Mantel-Cox log-rank test was used to compare survival rates between groups. For 1 recipient (no biopsy-confirmed AMR), no adequate material for urinary analysis of complement proteins was available. AMR, antibody-mediated rejection.

Study patients were followed for a median of 60 (IQR: 55–62) months. Overall, death-censored graft survival after index biopsy was 99% and 85% at 12 and 60 months, respectively. As shown in Figure 4, graft survival was inferior among patients with AMR, whereby graft loss rates were highest in patients with a C4d-positive AMR phenotype. No associations, however, were found for blood levels of C4d (Figure 4) and (data not shown) levels of C1q (*P* = 0.87), C3 (*P* = 0.76), C4 (*P* = 0.78), CH50 (*P* = 0.30), C3a (*P* = 0.70), and sC5b-9 (*P* = 0.37), respectively. In contrast, detection of C4d in urine was associated with inferior death-censored graft survival (*P* = 0.034), which paralleled a highly significant outcome effect of urinary protein excretion (Figure 4). Similar results were obtained for urinary C3a (*P* = 0.017) and C5a (*P*=0.019), while for sC5b-9 only a trend towards adverse survival was observed (*P* = 0.11) (data not shown). Bivariate Cox regression analysis revealed a significant outcome effect of proteinuria at screening (PCR <100 vs 100–1000 vs >1000 mg/g: HR: 3.99, 95%CI: 1.28-12.41, *P* = 0.017), however, failed to demonstrate an independent effect of urinary C4d (C4d detection vs no C4d detection: HR: 1.46, 95%CI: 0.35-6.16, *P* = 0.61). Similar results were obtained for urinary C3a and C5a, respectively (data not shown).

## DISCUSSION

A major result of this cross-sectional cohort study was that blood and urine markers of complement function and activation did not show any diagnostic value in conjunction with DSA detection late after kidney transplantation. Our detailed analysis of 83 stable DSA-positive recipients revealed that none of the tested parameters were able to identify ongoing AMR and thus dissect the pathogenicity and clinical relevance of a given HLA antibody pattern. There was even no relationship with capillary C4d deposition, which is known to specifically reflect local CP activation triggered by endothelium-bound DSA.^13,14,28^ A prominent finding, however, was that, in absence of any association with rejection, CP activation products in urine were tightly associated with levels of proteinuria (which were related to cg), indicating that their detection might rather nonspecifically mirror the extent of glomerular injury. While blood markers of complement were not associated with graft survival, univariate analysis revealed significant associations for complement detection in urine. However, bivariate analysis including proteinuria as a co-variable failed to demonstrate independent outcome effects.

A variety of experimental data and clinical observations are in support of a pathogenic role of the CP of complement as a trigger of rejection in organ transplantation,^11,29^ and this may provide a theoretical basis for the use of complement monitoring to dissect the relevance of a given DSA pattern. Complement activation triggered by preformed or de novo DSA may result in a sequence of events, including the release of anaphylatoxins (C3a, C4a, C5a), opsonin deposition (C3b, iC3b, and C3dg fragments), and in a final step, formation of the membrane attack complex (C5b-9). All these steps may promote the recruitment and activation of inflammatory cells, release of cytokines and chemokines, direct damage or activation (eg, upregulation of P-selectin or tissue factor) of the endothelium, vasodilation, and finally, activation of components of adaptive immunity.^11,29^ Several observations are in support of a role of CP activation in clinical transplantation. A prominent example is the finding of C4d staining in transplant capillaries, a marker of intra-graft CP activation found to strongly associate with graft survival.^14,28^ Moreover, detection of complement (C1q or C3d)-fixing DSA was reported to associate with rejection and graft failure,^15,30^ and there are data suggesting that complement-triggered transplant injury may be associated with a distinct pattern of gene expression that could even reflect the efficiency of complement inhibitory treatment.^31^

However, in recent years, there has been emerging evidence for a role of complement-independent injury mechanisms, in particular, Fc gamma receptor–mediated cellular activation.^12^ Strong support for complement independent mechanisms of injury come from microarray analysis of graft biopsies with subsequent pathway analysis which pointed to a dominant role of NK cell-triggered injury.^32^ Complement-independent transplant injury is supported by the frequent finding of C4d-negative phenotypes of rejection, especially in the late phase after transplantation.^33^ For example, in our cohort, only 21 of the biopsies were found to stain C4d positive, and, even when using a low threshold (minimal staining) for defining a positive staining, >50% of the AMR cases turned out to be C4d negative. In this respect, it may also be of interest that C4d itself was shown to exert distinct immunomodulatory effects, via interaction with atypical complement receptors on monocytes that might modulate the secretion of tumor necrosis factor-α or interleukin-6.^34^ The ability of antibodies to fix and activate complement, as can be quantified on HLA-coated microbeads, may critically depend not only on the distribution of involved immunoglobulin (sub)classes^35^ but also on antibody-binding strength, as to some extent reflected by the MFI recorded in bead assays,^9,36^ and one may speculate that the binding strength of a given HLA antibody may also directly relate to its ability to trigger complement-independent injury.

In this respect, an interesting finding was a prominent association of DSA MFI with AMR and C4d detection. As already reported in a previous study,^9^ the predictive accuracy of MFI even exceeded that of levels of complement fixation in different assay variants. As shown for capillary C4d, which was tightly related to DSA MFI (and complement-fixing ability), we found no association with complement split products in urine and blood. These data reinforce that measurement of complement in peripheral blood may not, as in other disease states (eg, autoimmune immune complex diseases^37^), mirror complement activation in affected tissue: in our patients, the transplant microvasculature. We have no good explanation for this observation, but in silent cases of late AMR, levels of complement consumption and levels of released split products may be too low to be visible in the circulation. The same was true for urinary complement patterns, perhaps also due to the fact that in AMR the endothelial surface, rather than the tubular system, is the primary site of antibody-antigen interaction. Moreover, urine levels of C activation products were a priori very often below the detection threshold, and there was a strong overlap of the effects of proteinuria which may have made the detection of subtle differences impossible. In this respect, a major finding was that applying bivariate Cox regression analysis, including protein excretion as a co-variable, we were unable to demonstrate an independent impact of urinary complement levels on death-censored graft survival.

In an earlier study, Hönger et al^17^ found that, as in our analysis, complement split product C4d in urine was tightly associated with proteinuria, but not with capillary C4d staining. These and our data support the view that complement product detection in the urine may be an unspecific measure of glomerular injury leading to protein excretion, independent of complement activation in the graft. At first sight, our data may be in contrast to a study by Bechtel et al,^16^ who found a relationship between urinary C4d (as well as ICAM-1 and VCAM-1) levels and rejection. However, levels were elevated particularly in patients with steroid-resistant rejection when compared with cases of steroid-sensitive or chronic rejection. They found no association at all for excretion of C5b-9; however, data on proteinuria were not provided.

Our data reinforce the need for defining innovative new approaches to improve noninvasive AMR monitoring. In the specific context of DSA monitoring, one promising example may be the detection of donor-derived cell-free DNA (dd-cfDNA), the level of which may reflect the extent of immunologic transplant injury.^38^ In a recent observational study of 90 clinically indicated biopsies obtained from 87 kidney transplant patients with a DSA result, Jordan et al^39^ found high positive and negative predictive values of dd-cfDNA (at a threshold of 1%) in relation to the diagnosis of active AMR, both exceeding 80%. The positive predictive value of DSA detection alone was only 48%, suggesting that dd-cfDNA evaluation may effectively supplement DSA testing as a noninvasive diagnostic tool.^39^

We are aware of the limitations of our study. One limitation may be that patient screening included only a single snapshot of testing without any information regarding the kinetics of complement components in relation to the clinical course and serial biopsy findings. Accordingly, using an arbitrary MFI threshold, our study design cannot exclude fluctuating DSA results in some of our patients (either in DSA-positive or DSA-negative subjects). Moreover, according to our screening protocol, only DSA-positive patients were considered for protocol biopsy, and therefore no data on the prevalence of rejection in absence of DSA were available, a possibility now considered in the revised Banff 2017 scheme.^2^ However, complement levels were similar in a group of matched DSA-negative patients, indicating that there might be no discrimination of DSA status. Our study focused on a cohort of patients who presented with stable graft function late after transplantation. According to defined inclusion criteria, subjects with acute deterioration of graft function and early cases of rejection were not included, and our results do not exclude some diagnostic value in more severe manifestations of acute AMR, in which CP activation may be more pronounced and perhaps visible in peripheral blood or urine. Finally, one may argue that even a detailed biopsy work-up, which was based on different diagnostic criteria, including molecular analysis, may not allow for a clear-cut differentiation between AMR and no AMR, even though this was supported by a substantial and highly significant difference with respect to molecular AMR scores. For example, for 3 of the 83 biopsies, no ultrastructural analysis was available, and in 1 case classified as no rejection, discrete cg visible by electron microscopy only cannot be excluded. Moreover, our biopsies were evaluated following the rules of the Banff 2013 scheme. More recently, AMR diagnosis was in some aspects modified in the Banff 2017 scheme,^2^ and a major modification may be that C4d staining or expression of validated transcripts/classifiers may now substitute for DSA. Following our study design, however, only DSA-positive cases were subjected to biopsies, and a possible role of DSA-negative AMR was not studied in our cohort.

The strengths of our study were a systematic prospective design, comparatively large number of cases recruited upon screening of >700 recipients, and a detailed characterization of DSA properties and biopsies.

Altogether, our study results indicate that noninvasive complement analysis in conjunction with DSA detection does not improve the diagnosis of silent AMR late after kidney transplantation. Our data reinforce the need for alternative concepts to noninvasively dissect the relevance of a given DSA pattern in this specific context.
